# Molecular characterization and PCR-based screening of *cry* genes from *Bacillus thuringiensis* strains

**DOI:** 10.1007/s13205-016-0583-7

**Published:** 2017-04-08

**Authors:** Devendra Jain, Sita D. Sunda, Suman Sanadhya, Dhruba Jyoti Nath, Sunil K. Khandelwal

**Affiliations:** 1 Department of Molecular Biology and Biotechnology, RCA, Maharana Pratap University of Agriculture and Technology, Udaipur, 313001 India; 2 Department of Soil Science, Assam Agricultural University, Jorhat, 785013 India

**Keywords:** *Bacillus thuringiensis*, Delta-endotoxin, *cry* genes, PCR, 16S rDNA, ARDRA, Screening

## Abstract

Novel *cry* genes are potential candidates for resistance management strategies, due to their different structures and modes of action. Therefore, it is desirable to clone and express novel *cry* genes from several new isolates of *Bacillus thuringiensis* (Bt). In the present study, 28 Bt strains were characterized at morphological and molecular level. All these strains are Gram positive, endospore forming and had shown different crystal morphologies when viewed under the microscope. The ARDRA (16S rDNA PCR-RFLP technique) with *Alu*I, *Hae*III, *Hinf*I and *Taq*I produced unique and distinguishable restriction patterns used for the molecular characterization of these isolates. Based on UPGMA clustering analysis, Bt strains showed significant molecular diversity and the dendrogram obtained differentiated 28 Bt strains into 1 major cluster at a similarity coefficient 0.56. PCR analysis demonstrated that the Bt strains showed diverse *cry* gene profiles with several genes per strain. The Bt strain G3C1 showed the presence of maximum *cry*-type genes by PCR. The toxicological characterization of these *cry* genes will have huge importance in transgenic technology and will be useful in transgenesis of crop plants for better resistance management.

## Introduction

*Bacillus thuringiensis* (Bt), is a Gram-positive, spore-forming soil bacterium that forms parasporal insecticidal crystal proteins during the stationary and sporulation phase of its growth cycle. These proteins are termed delta-endotoxins because of their intracellular location and have been used for many years as successful biological insecticides (Schnepf et al. 1998). Commercial Bt-based bio-insecticides used worldwide are applied at 10–50 g/acre or about 10^20^ molecules/acre, while chemical pesticides such as organophosphates and synthetic pyrethroids are applied about 8 × 10^24^ molecules/acre and 3 × 10^22^ molecules/acre, respectively. Thus, molecular potency of these toxins is 80,000 times better than organophosphates and 300 times greater than synthetic pyrethroids (Feitelson et al. 1992). Search for novel Bt strains may lead to the discovery of novel insecticidal proteins with higher toxicity which will be important for providing alternatives to cope up with the emergence of resistant insect populations. Therefore, there is a need of isolation of large number of Bt strains from diverse geographical conditions and cloning of many new types of insecticidal crystal proteins genes (Ramalakshmi and Udayasuriyan 2010).

The polymerase chain reaction (PCR) has been widely used to characterize the Bt strain collections (Ceron et al. 1995; Bravo et al. 1998; Ferrandis et al. 1999; Beron et al. 2005; Vidal-Quist et al. 2009). This technique is a highly sensitive method for rapid detection and identification of target *cry* gene sequences requires very small amounts of DNA and allows simultaneous screening of many Bt strains to classify them and predict their insecticidal activities. The efficacy of PCR in identifying the large family of *cry* genes is based on the presence of conserved regions. Another strategy for the screening is based on the multiplex PCR which uses more than two primers in a mixture of the same reaction (Juarez-Perez et al. 1997).

Continuous exposure to a single kind of Bt toxin can lead to resistance development in insects. Routine replacement of *cry* genes or pyramiding of *cry* genes could be useful for effective control of insect pests by Bt transgenic plants. Variation of a single amino acid can significantly influence the level of toxicity in Cry proteins (Udayasuriyan et al. 1994). The applications of Bt products as biopesticides are limited by their narrow host range, low toxicity to the targeted insects and the resistance from insects. Therefore, it is necessary to continuously screen novel *cry* genes and perform rational design based on a known Cry toxin (Lin et al. 2008). In general, the type of *cry* and *cyt* genes present in a strain correlates to some extent with its insecticidal activity (Porcar and Juárez-Pérez 2003). Since India is very rich in biodiversity and genetic resources, different Bt strains available in the country are valuable source for identification of indigenous, novel Bt genes, which could encode more potent toxins due to sequence variations (Jain et al. 2006). In the present study, 28 strains of Bt were characterized and screened for novel *cry* genes for better resistance management.

## Materials and methods

### Bacterial strains, chemicals and oligonucleotide primers

The Bt were isolated from the northeast region of India (Table 1). PCR chemicals, oligonucleotide primers and restriction enzymes were procured from Bangalore Genei, Pvt. Ltd., India, and AB genes Pvt. Ltd., UK. Microscopic examinations of the isolated strains were used for the characterization of Bt by physiological methods of staining such as Gram, endospore and crystal staining.

**Table 1:** Location of the Bt isolated from Assam region of India

Sl. no.	Location	Isolate code	Lat and long
1	Borbam tea estate	S20C4	*N* = 26°47.704; *E* = 94°31.868
2	Borbam tea estate	S22C1	*N* = 26°48.056; *E* = 94°31.801
3	Jogduwar, small tea garden	S29C2	*N* = 26°50.209; *E* = 94°26.551
4	Kharuapathar, rice field	D9C1	*N* = 27°51.35; *E* = 95°22.812
5	Borbam tea estate	S23C1	*N* = 26°48.056; *E* = 94°31.801
6	Borbam tea estate	S21C1	*N* = 26°47.704; *E* = 94°31.868
7	Borbam tea estate	S20C3	*N* = 26°47.704; *E* = 94°31.868
8	Gorjugonia forest	J11C1	*N* = 26°41.455; *E* = 94°08.605
9	Baruah bagan, tea garden	S11C1	*N* = 26°48.051; *E* = 94°33.483
10	Nabaruwara, rice field	T2C2	*N* = 27°74.633; *E* = 95°55.391
11	Nabaruwara, rice field	T5C1	*N* = 27°74.908; *E* = 95°56.913
12	Amguri, tea garden	S16C2	*N* = 26°48.494; *E* = 94°32.421
13	Charing, vegetable land	S6C3	*N* = 26°48.494; *E* = 94°32.421
14	Nabaruwara, rice field	T3C2	*N* = 27°74.369; *E* = 95°55.364
15	Hatigarh, rice field	T7C1	*N* = 27°62.579; *E* = 95°69.452
16	Hatigarh, rice field	T8C2	*N* = 27°62.606; *E* = 95°69.345
17	Khowang, rice field	D1C1	*N* = 26°27.278; *E* = 94°81.613
18	Tengakhat, rice field	D12C1	*N* = 27°41.047; *E* = 95°18.225
19	Charaibahi tea garden	J10C1	*N* = 26°40.893; *E* = 94°08.557
20	Sessa tiniali, rice field	D5C1	*N* = 27°25.843; *E* = 94°78.486
21	Borbam tea estate	S19C1	*N* = 26°47.704; *E* = 94°31.868
22	Sessa tiniali, rice field	D4C1	*N* = 27°55.792; *E* = 94°32.764
23	Nakhona gaon, rice field	S4C2	*N* = 26°55.792; *E* = 94°32.764
24	Golaghat, tea estate	G1C1	*N* = 26°63.334; *E* = 93°91.192
25	Nakhona gaon, rice field	S4C1	*N* = 26°55.792; *E* = 94°32.764
26	Gorjugonia forest	J11C3	*N* = 26°41.455; *E* = 94°08.605
24	Golaghat, rice fields	G2C1	*N* = 26.44788; *E* = 94.01534
28	Golaghat, vegetables	G3C1	*N* = 26.29748; *E* = 94.08121

### Molecular characterization using ARDRA of the 16S rDNA region

The total genomic DNA of Bt strains were isolated according to standard protocols (Kalman et al. 1993). The total DNA pattern of all Bt strains were analyzed on agarose gel. Genomic DNA isolated from Bt strains were used as template for the PCR amplification. Amplification of 16S rDNA region was performed with universal primers, 27F (5′AGAGTTTGATCCTGGCTCAG3′) and 1492R (5′ACGGCTACCTTGTTACGACTT3′). Each 40 µl reaction mixture contained 50 ng of genomic DNA of Bt strain, 50 ng of forward and reverse primers each, each dNTP at a final concentration of 200 µM and 1 U of Taq polymerase in 1× Taq buffer (with 15 µM MgCl_2_). Amplification was accomplished with the thermal cycler (Eppendorf, Germany). The PCR was performed with *Taq* polymerase for 30 cycles as follows: 94 °C for 1 min, 60 °C for 45 s and 72 °C for 1 min., the final extension was performed for 10 min at 72 °C. The 16S rDNA amplicons were digested using four different restriction endonucleases viz *Hinf*I, *Hae*III, *Alu*I and *Taq*I (Cihan et al. 2012). The restriction-digested products were analyzed on 2% agarose gel prepared in 1× TAE buffer containing 0.5 µg/ml of ethidium bromide. Electrophoresis was carried out at 100 V for 3 h in 1× TAE electrophoresis buffer. Data analysis was done using NTSYS-PC (Numerical Taxonomy and Multivariate Analysis System) software and SIMUQUAL Jaccard’s similarity coefficient (Rohlf 1997).

### PCR-based *cry* gene screening in indigenous Bt strains

Genomic DNA of the Bt was used in PCR with *cry* gene-specific screening primers (Table 2). The PCR was accomplished using an Eppendorf thermal cycler as prescribed by Jain et al. (2012).

**Table 2:** Oligonucleotide primers used for screening of partial *cry-*type genes

Sl. no.	Name	Sequence (5′→3′)	Amplification
1	Un1F	CATGATTCATGCGGCAGATAA AC	Partial *cry1* gene (277 bp)
2	Un1R	TTGTGACACTTCTGCTTCCCATT	
3	Un2F	GTTATTCTTAATGCAGATGAATGGG	Partial *cry2* gene (689–701 bp)
4	Un2R	CGGATAAAATAATCTGGGAAATAGT	
5	Un3F	CGTTATCGCAGAGAGATGACATTAAC	Partial *cry3* gene (589–604 bp)
6	Un3R	CATCTGTTGTTTCTGGAGGCAAT	
7	Un4F	GCATATGATGTAGCGAAACAAGCC	Partial *cry4* gene (439 bp)
8	Un4R	GCGTGACATACCCATTTCCAGGTCC	
9	Un5F	TTACGTAAATTGGTCAATCAAGCAAA	Partial *cry* 5, 12, 14, 21 genes (474–489 bp)
10	Un5R	AAGACCAAATTCAATACCAGGGTT	
11	Un7-8F	AAGCAGTGAATGCCTTGTTTAC	Partial *cry 7*–*8* gene (420 bp)
12	Un7-8R	CTTCTAAACCTTGACTACTT	
13	Un9F	CGGTGTTACTATTAGCGAGGGCGG	Partial *cry*9 genes (351–359 bp)
14	Un9R	GTTTGAGCCGCTTCACAGCAATCC	
15	Un11F	TTCCAACCCAACTTTCAAGC	Partial *cry*11 genes (305 bp)
16	Un11R	AGCTATGGCCTAAGGGGAAA	
17	VipF	CCTCTATGTTGAGTGATGTA	Partial *vip3* genes (1000 bp)
18	VipR	CTATACTCCGCTTCACTTGA	
19	Cyt1F	AACCCCTCAATCAACAGCAAGG	Partial *cyt1* genes (522–525 bp)
20	Cyt1R	GGTACACAATACATAACGCCACC	
21	Cyt2F	AATACATTTCAAGGAGCTA	Partial *cyt2* genes (469 bp)
22	Cyt2R	TTTCATTTTAACTTCATATC	

## Results and discussion

### Characterization of indigenous Bt strains

All the Bt isolates were found to be Gram positive and endospore forming. Similarly different crystal morphologies were seen blue under the white background and showed bi-pyramidal, spherical, cuboidal, rectangular, irregular crystals, etc. The shape of the crystalline inclusions varied among the 28 Bt strains. Shishir et al. (2012) identified Bt isolates based on their hemolytic activity, presence of parasporal crystal proteins and crystal protein profile and observed five different types of parasporal crystal proteins such as spherical, bi-pyramidal, irregular pointed, cuboidal and irregular shaped which formed irregular white colonies with a pink background among the isolates which indicates the diversity of the local Bt isolates. Similarly, Unalmis et al. (2015) isolated Bt-like colonies and characterized them on the basis of Gram staining, spore straining and crystal staining. From 60 bacterial colonies observed through bright field microscopy, 28 isolates were identified as Bt based on the presence of crystalline inclusions.

### Molecular characterization of indigenous Bt strains using ARDRA

**Fig. 1:**
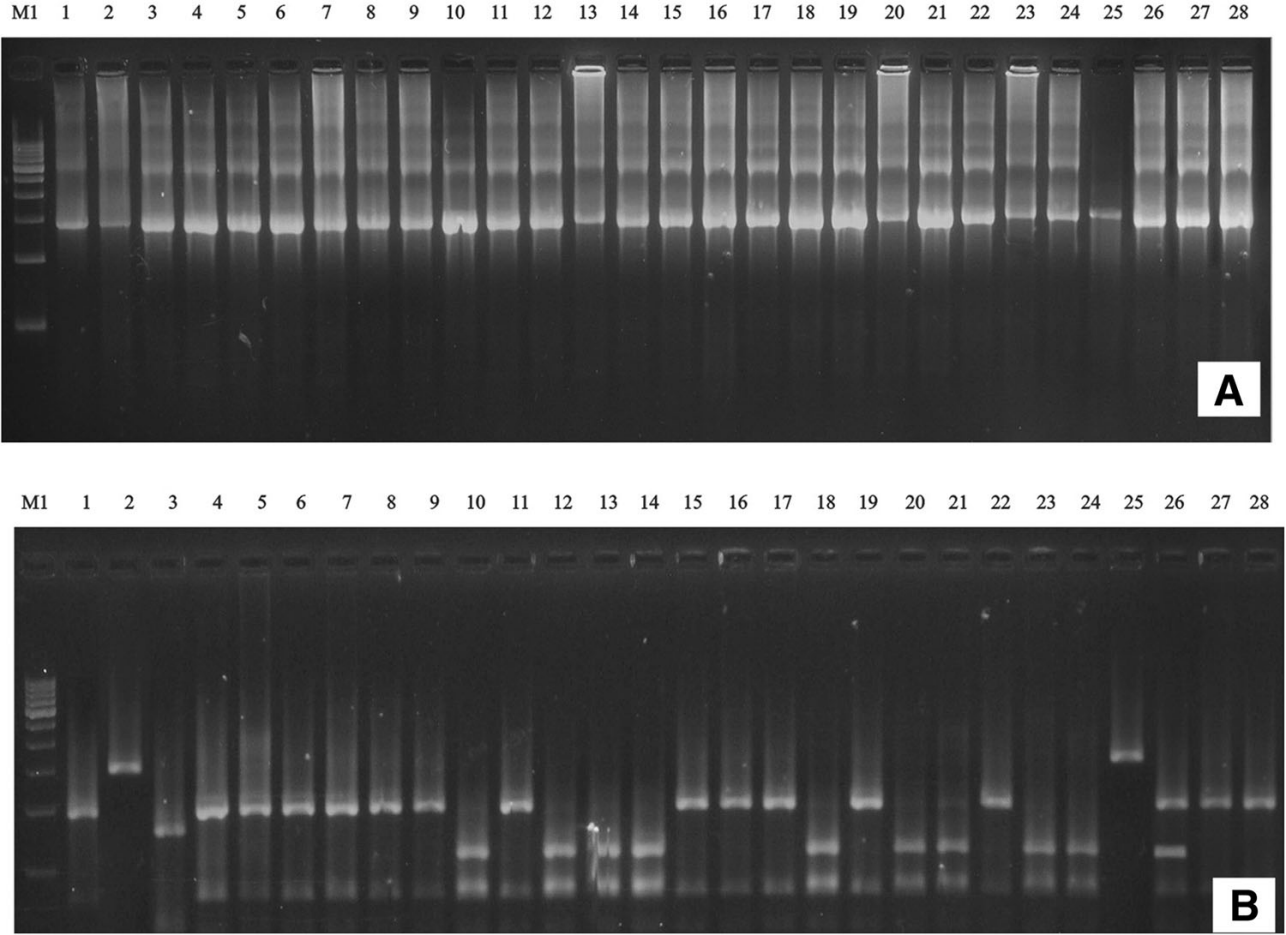
Agarose gel electrophoresis of **a** PCR amplification of 16S rDNA region from native Bt strains. **b** ARDRA patterns of 16S rDNA sequence of Bt strains by restriction enzyme *AluI*. *M1* 500 bp DNA ladder, Bt strains *1* S4C1, *2* S16C2, *3* S4C2, *4* D4C1, *5* T5C1, *6* S20C4, *7* G1C1, *8* S19C1, *9* S22C1, *10* S20C3, *11* D9C1, *12* T8C2, *13* T2C2, *14* S21C1, *15* D1C1, *16* G3C1, 1*7* J11C1, *18* D12C1, *19* T3C2, *20* G2C1, *21* D5C1, *22* S29C2, *23* J11C3, *24* T7C1, *25* S23C1, *26* S6C3, *27* S11C1, *28* J10C1

Amplified rDNA (Ribosomal DNA) restriction analysis (ARDRA) is the extension of the technique of RFLP (restriction fragment length polymorphism) to the gene encoding the small ribosomal subunit (16S) of bacteria. The technique involves an enzymatic amplification using primers directed at the conserved regions at the ends of the 16S rDNA, followed by digestion using restriction enzymes. The pattern obtained is said to be representative of the species analyzed and important for their molecular characterization. In the present study ARDRA produced a fingerprint based on length polymorphism for molecular characterization of native Bt strains. Four restriction endonucleases viz., *Hinf*I, *Hae*III, *Alu*I and *Taq*I were used for restriction fragment analysis of amplified 16S rDNA. The banding patterns of the representative Bt are shown in Fig. 1, with standard molecular weight marker. Totally 20 bands of varying sizes were observed in all the 28 strains when digested with four restriction enzymes. When digested with *Hae*III, 7 different DNA fragments were obtained, whereas 3 different DNA fragments were obtained with *Hinf*I endonuclease, *Taq*I digestion resulted 6 different DNA fragments while *Alu*I resulted in 4 different DNA fragments.

Genetic similarity estimates the variations based on ARDRA banding patterns, which were calculated using method of Jaccard’s coefficient analysis. The similarity coefficient matrix generated was subjected to algorithm “Unweighted Pair Group Method for Arithmetic Average (UPGMA)” to generate clusters using NTSYS 2.02 pc program. The pairwise comparison of ARDRA patterns based on both shared and unique amplification products was made to generate a similarity matrix. Similarity indices established on the basis 20 bands of 4 restriction enzymes ranged from 0.11 to 0.93.

**Fig. 2:**
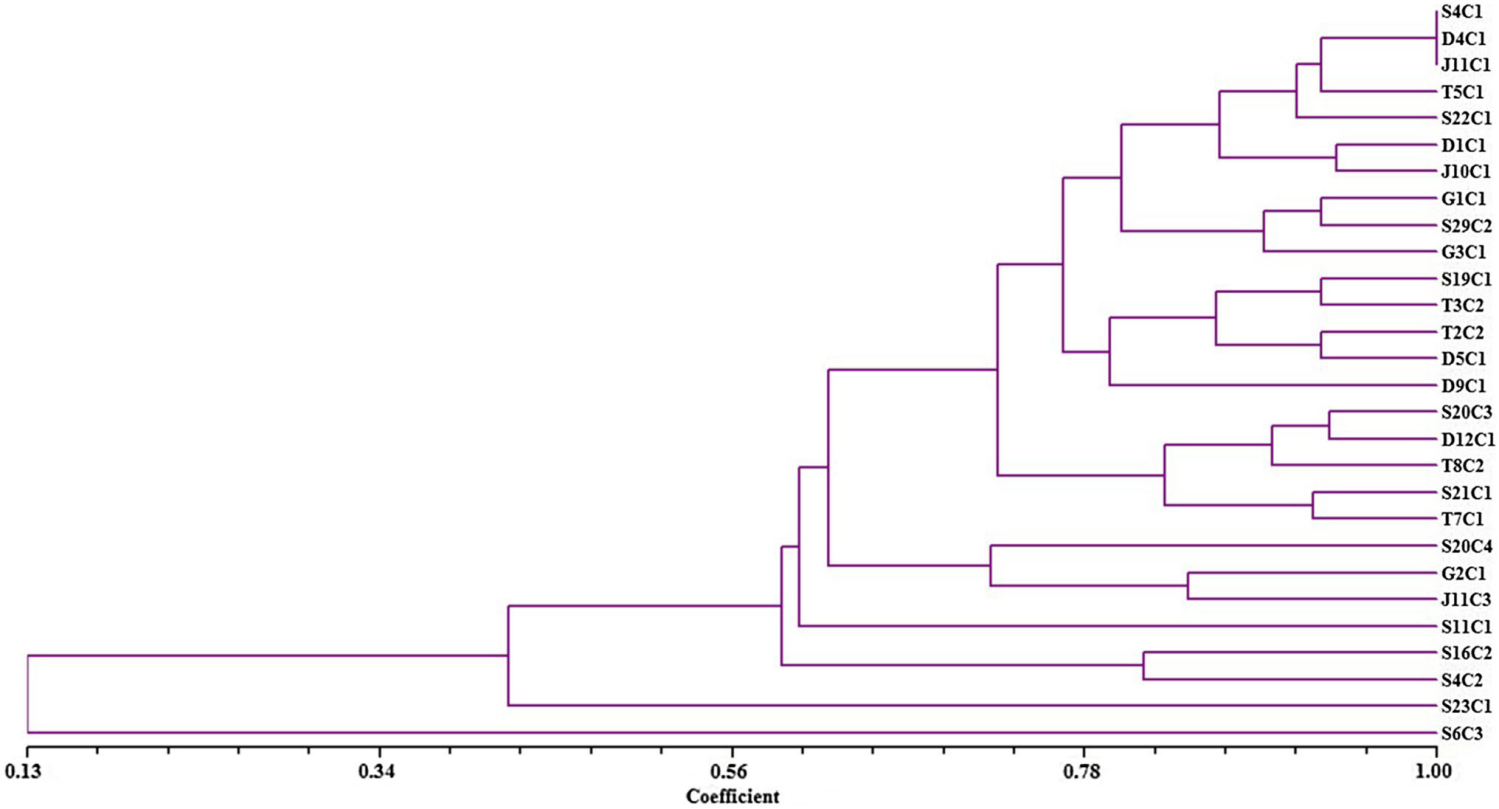
Dendrogram based on ARDRA pattern of 28 Bt isolates

The dendrogram (Fig. 2) is a close representation of the values obtained in the similarity matrix. The dendrogram depicted the relationship among the Bt strains and clearly divided into one major cluster at a similarity coefficient 0.56. The dendrogram clearly indicated that S6C3 and S23C1 were different from remaining strains, hence these are more diverse. The first (A) cluster was divided into two sub-clusters A1 and A2. Sub-cluster A1 included 24 strains and further divided into two sub-clusters A1a and A1b. Sub-cluster A1a included strains J11C3, G2C1, S20C4, T7C1, S21C1, T8C2, D12C1, S20C3, D9C1, D5C1, S22C1, T5C1, J11C1, D4C1, and S4C1. S4C1, D4C1 and J11C1 and sub-cluster A1b comprises only one strain S11C1. Sub-cluster A2 comprises 2 strains S16C2 and S4C2.

Molecular techniques have helped to develop easy and rapid methods to perform microbial characterization at genus, species and even at strain level. Molecular markers have been found to be strain specific and have proved to be valuable tools in the characterization and evaluation of genetic diversity within and between species and populations. Identification of *Bacillus* species using conventional sequencing methods can divulge their taxonomic affiliation, but there are certain groups of *Bacillus* where alternate methods like ARDRA and PCR fingerprinting can expose the exact lineage of the species rapidly.

Saadaoui et al. (2009) reported a new *Bacillus thuringiensis* kurstaki strain BLB1, isolated from a Tunisian soil sample showed the same 16S rDNA ARDRA profile than HD1 using *Taq*I, *Alu*I, *Mbo*II, and *Msp*I restriction enzymes. Gowdaman et al. (2014) reported that a collection of 171 soil bacterial isolates was analyzed for the occurrence of genus *Bacillus* using group-specific primers and ARDRA was performed for the *Bacillus*-positive isolates with standard *Bacillus* strains. Sangeetha et al. (2016) analyzed 15 bacterial isolates by ARDRA and used eight restriction endonucleases (*Cfo*I, *Hinf*I, *Rsa*I, *Dde*I, *Sau3A*I, *Alu*I, *Hae*III, and *Msp*I) forming two heterogenous main clusters after analysis by unweighted pair-group method using arithmetic averages.

### PCR-based screening of *cry* genes in native Bt strains

**Fig. 3:**
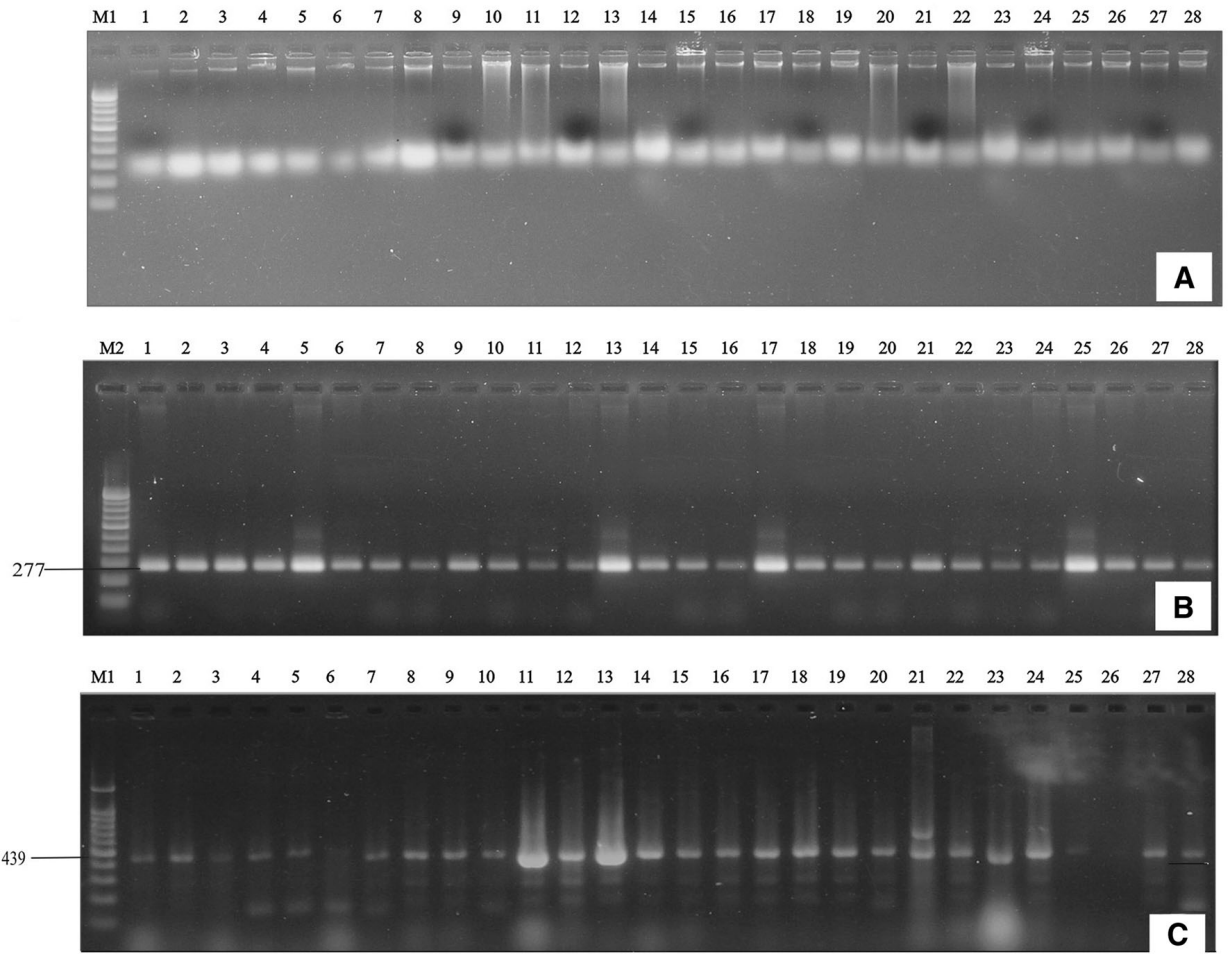
Agarose gel electrophoresis of **a** DNA isolation from native Bt strains. **b** PCR amplification of partial *cry* 1 gene from Bt strains. **c** PCR amplification of partial *cry4* gene from Bt strains. *M1* 500 bp DNA ladder, Bt strains *1* S4C1, *2* S16C2, *3* S4C2, *4* D4C1, *5* T5C1, *6* S20C4, *7* G1C1, *8* S19C1, *9* S22C1, *10* S20C3, *11* D9C1, *12* T8C2, *13* T2C2, *14* S21C1, *15* D1C1, *16* G3C1, 1*7* J11C1, *18* D12C1, *19* T3C2, *20* G2C1, *21* D5C1, *22* S29C2, *23* J11C3, *24* T7C1, *25* S23C1, *26* S6C3, *27* S11C1, *28* J10C1

Amplification of expected size of PCR products in different primer pairs (Fig. 3) indicated the presence of the above-mentioned *cry-*type genes in Bt strains (Table 3). The primers *cry2, cry3, cry5* and *cry7*–*8* did not show any amplification in PCR-based *cry* gene screening. The strains containing *cry*1-type genes were the most abundant (100%) in the indigenous Bt strains since all the strains were harboring these genes followed by *cry*4 (84.14%), *cry9* (64.28%), *cry11* (39.28%), *cyt2* (39.28%), and *vip3A* (25%) (Table 4). Agarose gel electrophoresis showed non-specific amplification along with specific partial *cry* gene amplicon which was also observed in many published research findings using the same screening primers.

**Table 3:** Distribution of *cry* and *cyt-*type genes in native Bt isolates

Genes	1	2	3	4	5	6	7	8	9	10	11	12	13	14
*cry1*	+	+	+	+	+	+	+	+	+	+	+	+	+	+
*cry 2*														
*cry 3*														
*cry4*	+	+	+	+		+	+	+		+	+	+	+	+
*cry 5*														
*cry 7*–*8*														
*cry9*				+	+	+				+	+	+	+	+
*cry11*				+		+			+		+	+		+
*cyt1*				+			+		+	+	+	+		
*cyt2*						+				+	+	+	+	
*Vip*	+		+							+	+			

Genes	15	16	17	18	19	20	21	22	23	24	25	26	27	28
*cry1*	+	+	+	+	+	+	+	+	+	+	+	+	+	+
*cry 2*														
*cry 3*														
*cry4*	+	+	+	+	+	+	+		+	+	+	+		
*cry 5*														
*cry 7*–*8*														
*cry9*		+	+		+	+		+	+	+	+		+	+
*cry11*		+	+		+		+				+			
*cyt1*											+		+	+
*cyt2*		+	+	+	+			+	+					
*Vip*			+					+					+	

+ indicates the presence of *cry* gene by PCR

Bt strains 1–28 represent 1. D1C1, 2. T7C1, 3. J11C1, 4. S6C3, 5. T8C2, 6. S19C1, 7. S23C1, 8. T5C1, 9. S4C1, 10. S20C3, 11. G3C1, 12. J11C3, 13. G2C1, 14. S29C2, 15. T3C2, 16. S11C1, 17. D4C1, 18. S21C1, 19. D5C1, 20. J10C1, 21. G1C1, 22. D9C1, 23. D12C1, 24. S22C1, 25. S20C4, 26. S16C2, 27. T2C2 and 28. S4C2

**Table 4:** Frequency of *cry-*, *cyt-* and *vip-*type genes in Bt strains

Genes	Frequency (%)
*Cry 1*	100
*Cry 4*	82.14
*Cry 9*	64.28
*Cry 11*	39.28
*Cyt 1*	32.14
*Cyt 2*	39.28
*Vip*	25

Jain et al. (2012) observed the frequency of *cry-*type genes in eight Bt strains (IS1–IS8) with the result that the *cry*1 type genes were most abundant in the indigenous isolates since all the strains were harboring these genes, followed by *vip*3A (87.5%), *cry*2 (75%), *cry*9 (62%), *cry3* (50%), *cry*11 (37.5%), *cry*7–8 (37.5%), *cry*5, 12, 14, 21 (25%), *cyt1* (25%), *cry*4 (12.5%) and *cyt*2 (12.5%) as detected by PCR. Patel et al. (2012) reported the diversity of *cry* genes from different soil types and climatic environments and reported the presence of *cry1, cry2, cry3, 7, 8, cry4, cry5, 12, 14, 21, cry11, cry13* and *cyt1* genes from Bt, whereas absence of *cry3* and *cry13* genes were reported in the isolates of non-agricultural samples. Similarly, Salekjalali et al. (2012) identified isolates harboring different *cry*-type genes through PCR and found 47% of the strains amplified with the *cry1* primer, 29% with *cry3* and 13% with *cry4.* Salama et al. (2015) reported that *cry*1 gene is the most abundant in these isolates (83.33%) among tested *cry*-type genes, followed by *cry*1 gene subfamilies (*cry1B* and *cry1C*) with percentage of 38.88 and 77.77%, respectively. The tested isolates showed the presence of *cry2A* gene, but not all of these isolates were positive for *cry*2 gene (55.55%). Only 27.77 and 16.66% of the tested isolates harbor *cry*4 and *cry3* genes, respectively.

The use of PCR has greatly improved *cry* gene detection; however, this method is mostly limited to members of previously described gene families and requires a large number of primers. The results of the present study suggest the presence of diversity in the native Bt isolates. Further studies on cloning and characterization of those novel *cry* genes from these new isolates of Bt will be useful and open new opportunities in the area of integrated pest management for sustainable agriculture.

## Abbreviations

Bt: *Bacillus thuringiensis*
Cry ptrotein: Crystal protein
PCR: Polymerase chain reaction
ARDRA: Amplified ribosomal DNA restriction analysis
UPGMA: Unweighted pair-group method for arithmetic average
